# Isomorphs in nanoconfined liquids†

**DOI:** 10.1039/d1sm00233c

**Published:** 2021-09-13

**Authors:** Benjamin M. G. D. Carter, C. Patrick Royall, Jeppe C. Dyre, Trond S. Ingebrigtsen

**Affiliations:** H.H. Wills Physics Laboratory Tyndall Avenue Bristol BS8 1TL UK; Bristol Centre for Functional Nanomaterials Tyndall Avenue Bristol BS8 1TL UK; School of Chemistry, University of Bristol Cantock's Close Bristol BS8 1TS UK; Centre for Nanoscience and Quantum Information Tyndall Avenue Bristol BS8 1FD UK; Department of Science and Environment, Roskilde University Postbox 260 DK-4000 Roskilde Denmark trond@ruc.dk

## Abstract

We study in this paper the possible existence of Roskilde-simple liquids and their isomorphs in a rough-wall nanoconfinement. Isomorphs are curves in the thermodynamic phase diagram along which structure and dynamics are invariant in suitable nondimensionalized units. Two model liquids using molecular dynamics computer simulations are considered: the single-component Lennard-Jones (LJ) liquid and the Kob–Andersen binary LJ mixture, both of which in the bulk phases are known to have good isomorphs. Nanoconfinement is implemented by adopting a slit-pore geometry with fcc crystalline walls; this implies inhomogenous density profiles both parallel and perpendicular to the confining walls. Despite this fact and consistent with an earlier study [Ingebrigtsen *et al.*, *Phys. Rev. Lett.*, 2013, **111**, 235901] we find that these two nanoconfined liquids have isomorphs to a good approximation. More specifically, we show good invariance along the isomorphs of inhomogenous density profiles, mean-square displacements, and higher-order structures probed using the topological cluster classification algorithm. Our study thus provides an alternative framework for understanding nanoconfined liquids.

## Abstract 2

Invariance of dynamics along an isomorph (left) compared to an isochore (right) in a nanoconfined liquid.

## Graphical abstract

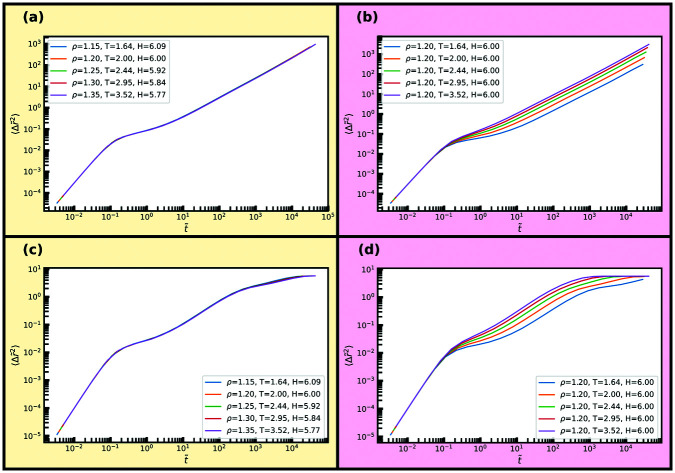

## I. Introduction

An important simplification in the study of liquids *via* computer simulations is to apply so-called periodic boundary conditions. The liquid is thereby free from any confining surfaces which affect its structure and dynamics by imposing an external force field on the liquid.^1,2^ This simplification is, however, hard to achieve in experiments, and most liquids in nature are in contact with, or confined by, one or several surfaces.^3–14^ Recent experiments on levitation of metallic liquids using electrostatic or magnetic fields,^15–18^ ionic solution droplets in optical tweezers,^19^ and especially colloids^20^ come closer to the simplification of standard computer simulations,^15–18^ but still have “free surfaces” that may affect the probed quantities. Significantly, the added complexity induced by the walls has made fundamental theories of nanoconfined liquids slower to develop, in particular for the dynamics.^21–26^

Roskilde-simple liquids (also called R-simple liquids) are liquids with strong correlations between equilibrium fluctuations of the virial *W* and the potential energy *U* in the *NVT* ensemble.^27–34^ Van der Waals and metallic liquids have been shown to belong to this class of liquids whereas, *e.g.*, hydrogen-bonding liquids are not R-simple. R-simple liquids have isomorphs in the thermodynamic phase diagram which are curves along which structure and dynamics are invariant in reduced units. This fact makes R-simple liquids simpler than other types of liquids. As an example, Rosenfeld's excess entropy scaling can be explained using the concept of isomorphs.^35,36^ In Rosenfeld's excess-entropy scaling reduced transport coefficients *X̃* are functions of the entropy minus the ideal contribution at the same density and temperature, *i.e.*, *X̃* = *f*(*S*_ex_), where *S*_ex_(*ρ*,*T*) = *S*(*ρ*,*T*) − *S*_id_(*ρ*,*T*). Since both the reduced dynamics and the excess entropy are invariant along the same curves (isomorphs), this fact explains Rosenfeld's excess entropy scaling. This scaling law is, however, only one of many consequences of having isomorphs (see, *e.g.*, ref. 32).

Extending the isomorph theory to nanoconfined fluids is therefore of paramount importance as this would offer an alternative framework in which confined fluids could be understood and analyzed. An earlier computer simulation study investigated R-simple liquids in confinement using an idealized slit-pore geometry.^37^ It was found that even heavily nanoconfined liquids have isomorphs to a good approximation, except for confinements around one or two particle diameters. Idealized slit-pore confinement implies that only the density profile perpendicular to the walls is inhomogenous.

To model more realistic confinement conditions, we study in this paper two model liquids confined to a slit-pore geometry with fcc crystalline walls. The structure and dynamics of liquids confined by fcc walls have been studied before and shown to exhibit density profiles that are highly inhomogenous, both parallel and perpendicular to the confining walls.^3–5^ Our aim here is to investigate whether isomorphs survive under such strong inhomogeneities. This is a first step in the direction of studying more realistic confinement conditions relevant, *e.g.*, for industrial applications and biological systems.

We find that, despite the appearance of strong inhomogenous density profiles in the liquid, isomorphs do survive down to a few particle-diameters confinements enabling the applicability of results from the isomorph theory to more complex confined liquids. We conjecture from this study that R-simple liquids and isomorphs are relevant for a much larger class of confinements consistent with previous studies of excess-entropy scaling in nanoconfinement.^38^

At lower temperatures than what we consider here, higher-order structures have been shown to exhibit differences between isomorphic states.^39^ Here therefore, we also probe measures of higher-order structures and investigate minimum energy clusters in the liquid of interest.^40^ We find that little difference is seen in higher-order structure along isomorphs of the confined liquids.

The paper is organised as follows. Section II introduces the models and methods we apply in this study, and Section III gives a short introduction to R-simple liquids and their isomorphs. Section IV presents results for the single-component Lennard-Jones (LJ) liquid where we, amongst other things, study isomorphs. Section V presents similar results for the Kob–Andersen binary LJ mixture. Section VI summaries and presents a brief outlook.

## II. Simulation methods

We use standard Nosé–Hoover molecular dynamics computer simulations in the *NVT* ensemble (as implemented in the RUMD package^41^) to study two model liquids in confinement: the single-component Lennard-Jones (SCLJ) liquid and the Kob–Andersen binary LJ mixture (KABLJ).^42,43^ In both models, the pair interaction between the liquid particle *i* of type α and the liquid particle *j* of type β is described by the LJ pair potential given by1
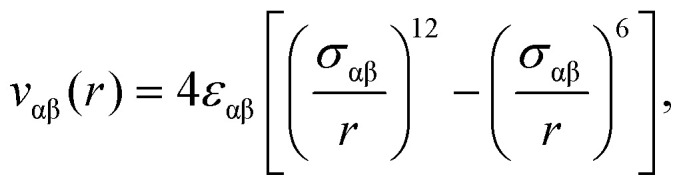
where *ε*_αβ_ is the strength of the pair interaction (α or β is equal to type A or B for KABLJ), *r* is the distance separating the particles, and *σ*_αβ_ is the separation distance at which the pair potential is zero. For the LJ model we have *ε*_AA_ = 1, *σ*_AA_ = 1, and *m*_A_ = 1 (*m* is the particle mass), whereas the KABLJ mixture has *σ*_AA_ = 1, *ε*_AA_ = 1, *σ*_AB_ = 0.80, *ε*_AB_ = 1.5, *σ*_BB_ = 0.88, and *ε*_BB_ = 0.5. The masses of both particles in the KABLJ model are set to unity. The pair potential is truncated-and-shifted at the distance *r*_c_ = 2.5*σ*_αβ_.

For most state points we simulate around one million time steps with a time step of Δ*t* = 0.0025 after obtaining equilibrium. Equilibrium is ascertained from the decay of the intermediate scattering function and by running the simulations back-to-back at least twice.

### A. Simulation units

Throughout the study, we use two different sets of nondimensionalized units: one set is based on the microscopic parameters of the LJ potential with length scale *σ*_AA_ and energy scale *ε*_AA_ of the larger (A) particle, which is standard in computer simulations, and another set of nondimensionalized units using macroscopic quantities with length given in units of *ρ*^−1/3^, energy in units of *k*_B_*T*, and time in units of 
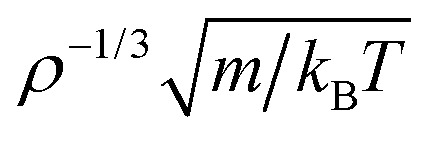
 as applied in isomorph scaling.^30^ We refer to macroscopic nondimensionalized units as reduced units and use a tilde above the variable name to indicate a reduced quantity; otherwise LJ units are implicitly assumed.

### B. Nanoconfinement

**Fig. 1:**
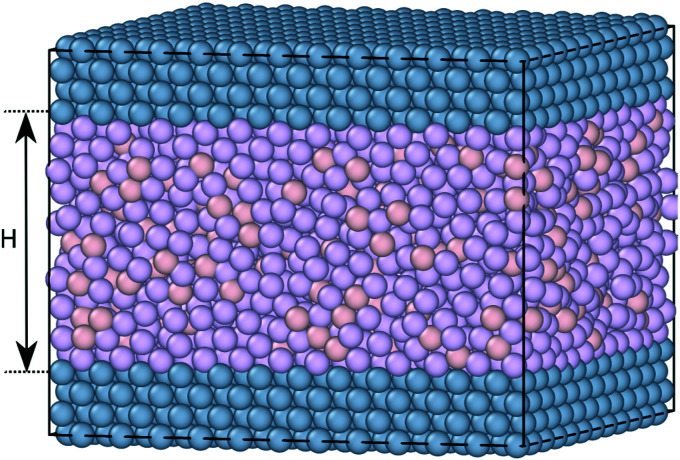
The simulated slit-pore geometry confinement for the KABLJ mixture.^44^ Dark purple is the larger A-particle, pink is the smaller B-particle, and blue is the crystalline wall particles. The distance between the two walls is *H*, here *H* = 8. The two crystal planes are in registry.

Nanoconfinement is modelled using a slit-pore geometry in which the 100 surface of a fcc crystal is exposed to the liquid. The two crystal planes are placed in registry (*i.e.*, the two crystal planes do not match). The distance between the two walls is denoted by *H*, measured from the centers of the confining fcc particles (see Fig. 1).

The slit pore is created as follows. First a fcc crystal with the correct density (using four crystal layers) is created and then replicated and translated a large distance away. Another fcc lattice is then created with the correct number of liquid particles in between the two walls for the chosen liquid density and wall spacing. The system is then compressed very slowly while the liquid particles are allowed to melt. In doing so, standard periodic boundary conditions are applied in the *z*-direction but since the liquid particles cannot penetrate through the walls, and the walls are frozen in place (see below) the simulations mimic infinite confinement.

The liquid–wall pair interactions are described by the LJ potential in eqn (1). For the SCLJ liquid we use the parameters *σ*_AW_ = 1 and *ε*_AW_ = 1, in which W denotes a wall particle. For the KABLJ mixture we derived the interaction parameters from Lorentz–Berthelot mixing rules with *σ*_AW_ = 0.97, *ε*_AW_ = 1, *σ*_BW_ = 0.91, and *ε*_BW_ = 0.707, where we used *σ*_WW_ = 0.94 and *ε*_WW_ = 1 to calculate these numbers. The cutoff of the liquid–wall pair interaction is *r*_c_ = 2.50*σ*_*α*W_.

For the SCLJ liquid we use the liquid density *ρ* = 0.85 as a reference and vary the temperature *T* = [0.50; 6] in steps of Δ*T* = 0.2 with *H* = [2, 4, 6, 8, 10] and *N* = [274, 1233, 822, 1024, 1370] liquid particles and *N* = [1024, 2304, 1024, 1096, 1024] wall particles. The same values are used to vary *ε*_AW_ in steps of Δ*ε*_AW_ = 0.2 at *T* = 2. The density of the fcc walls is for the majority of the simulations (except for the isomorph generation) kept fixed at *ρ*_W_ = 1, but we also study the effect of varying this parameter at *H* = 6 and *T* = 2 for SCLJ. For *ρ*_W_ = 0.4, 0.6, 0.8, 1.2, 1.4 we used *N* = [3408, 2601, 2147, 1638, 1478] liquid particles and for the wall *N* = 2304.

For the KABLJ mixture we focus on the liquid density *ρ* = 1.20 with *T* = [0.5; 6] in steps of Δ*T* = 0.2 with *H* = [4, 6, 8, 10] and *N* = [2477, 2090, 2787, 3484] liquid A particles and *N* = [4096, 2304, 2304, 2304] wall particles. The same values are used to vary *ε*_αW_ in steps of Δ*ε*_*α*W_ = 0.2 at *T* = 2.5. The density of the wall is *ρ*_W_ = 1, and we use a single-component crystal.

The simulations use a Nosé–Hoover *NVT* thermostat on the liquid particles whereas, for simplicity, the wall particles are frozen in place, *i.e.*, *T* = 0 for the walls. Thermostatting the walls and the nature of the exposed surface may have observable effects on the structure and dynamics of confined liquids.^45,46^ This issue we ignore in this investigation.

The phase behavior of confined liquids is rather complex and has been studied extensively in the literature.^3–5,13,47–50^ For confined systems the solid–liquid transition is sensitive to the spacing between the walls *H*, *e.g.*, how many crystal layers the pore can accommodate, the registry of the two walls, and the strength of the wall–liquid interaction. This transition has also been shown to occur as several transitions in a cascade-like way depending on *H*.^49^ Furthermore, precrystallization in the layer closest to the walls is common in confinement; in our case where the walls are fcc this phenomenon appears already at high temperatures for both liquids.

### C. Topological cluster classification

We identify higher-order structures of the confined liquid using the topological cluster classification (TCC) algorithm which carries out a Voronoi decomposition and seeks structures topologically identical to geometric motifs of particular interest.^40^ In Sections IV and V, we also compare results of the TCC for the confined system with the bulk system at the same density and temperature. For these bulk simulations we use standard Monte-Carlo NVT simulation with *N* = 4000 particles.

## III. Roskilde-simple liquids

We provide here a brief introduction to Roskile-simple (R-simple) liquids and their isomorphs; a review is given in ref. 32. R-simple liquids are characterized by strong correlations between the equilibrium fluctuations of the potential energy *U* and virial *W* in the *NVT* ensemble.^27–34^ This correlation is quantified by the Pearson correlation coefficient *R* defined by^27^2
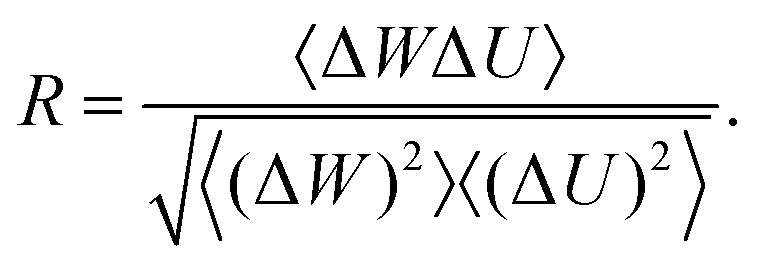
Here Δ denotes deviation from the average value, and the averages are taken in the *NVT* ensemble (*i.e.*, canonical ensemble averages). R-simple liquids are those for which the correlation coefficient *R* is above 0.90; the value of *R* depends on the state point. The correlation coefficient has been shown to be high in large parts of the phase diagram for many systems, typically in the condensed liquid and solid phases, but not in the gas phase.

Van der Waals and metallic liquids are usually R-simple whereas, *e.g.*, hydrogen-bonding, covalent-bonding and strongly ionic liquids are not. More specifically, in simulations the SCLJ liquid, the KABLJ mixture, the Wahnström OTP model, bead-spring polymer models, and many more are R-simple. Strong *UW* correlation has also been verified in experiments on weakly dipolar organic molecules.^51–53^

Initially, the bulk liquid phase was studied, but the concept of R-simple liquids was later extended to crystals,^54^ nanoconfined liquids,^37^ nonlinear sheared liquids,^55^ polydisperse liquids,^34,56^ quantum-mechanical *ab inito* liquid metals,^57^ and more.^58^

R-simple liquids are characterized by the following ordering of potential energy values for most configurations^33^

3 *U*(**R**_a_) < *U*(**R**_b_) ⇒ *U*(*λ***R**_a_) < *U*(*λ***R**_b_),

where **R**_a_ and **R**_b_ are 3N-dimensional configurational-space vectors of a given density, and *λ* is a factor scaling uniformly these configurations to a new density.

R-simple liquids exhibit a number of simple properties,^59^ most of which are consequences of the existence of isomorphs. Isomorphs are curves in the thermodynamic phase diagram of R-simple liquids along which structure and dynamics to a good approximation are invariant in reduced units (see the previous section). Isomorphs are defined as curves of constant excess entropy and can be generated *via* the following relation4
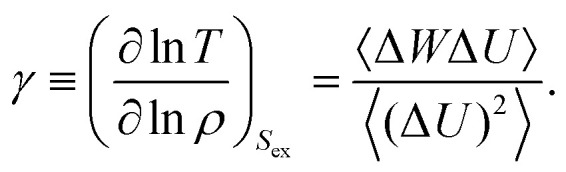
This is a general thermodynamic relation in the *NVT* ensemble.^30,60^ The parameter *γ* is called the density-scaling exponent because it is a key quantity when applying density scaling.^30,61,62^ The procedure to generate an isomorph in simulations using the above relation is as follows: a simulation is performed at a given state point, *γ* is calculated, a new slightly higher or lower density is chosen, and from discretization of eqn (4) the new temperature is calculated.

In this article, however, we generate isomorphs using a different procedure. A first-order approximation to eqn (3) implies that the Boltzmann factors of two isomorphic state points are proportional (also the old definition of isomorphs^30^), *i.e.*,

5 exp(−*U*(**R**^(1)^)/*k*_B_*T*_1_) = *C*_12_ exp(−*U*(**R**^(2)^)/*k*_B_*T*_2_),

where *C*_12_ is a constant, and the comparison is performed for configurations at densities *ρ*_1_ and *ρ*_2_ for which *ρ*_1_^1/3^**R**^(1)^ = *ρ*_2_^1/3^**R**^(2)^, *i.e.*, having the same reduced coordinates. From this equation it follows that if a simulation is performed at density *ρ*_1_ and temperature *T*_1_ and configurations are scaled uniformly to a different density *ρ*_2_ at which the potential energy is evaluated, the linear regression slope of *U*_2_*vs. U*_1_ provides the ratio of the temperatures *T*_2_/*T*_1_ of the two isomorphic state points. This is called the direct isomorph check.^30^ In this article we change density in steps of approximately 5%.

For confined systems the wall distance *H* is an independent variable, similar to density and temperature in bulk liquids. We choose here to let *H* (and the crystal density) follow the overall scaling in liquid density, *i.e. H* scales with (*ρ*_1_/*ρ*_2_)^1/3^. This choice is consistent with the definition in a previous study of isomorphs in nanoconfinement.^37^ This study^37,63^ also indicated that it might be possible to keep the wall distance fixed, but we do not consider this in more detail here. It is important to note that the state points we identify as being isomorphic under confinement in general are not isomorphic in the bulk.

As mentioned in Section II, precrystallization near the walls is well known for nanoconfined liquids. Previous works^49,50^ on confined liquids have shown that it can be advantageous to exclude these particles in the free energy analysis as they effectively become part of the walls. This might lead to the question of whether or not this should be taken into account in the generation of isomorphs. We found in a previous study^63^ that this crystallization scales perfectly along the isomorphs and hence one does not need to exclude them.

## IV. Single-component Lennard-Jones liquid

We commence the study by probing the correlation coefficient *R* and density-scaling exponent *γ* for the SCLJ liquid in confinement. The next section considers the same quantities for the KABLJ mixture.

### A. Variation in the correlation coefficient *R* and density-scaling exponent *γ*

This subsection studies how the above mentioned quantities are affected by changing the following parameters related to the confinement: the distance between the two walls *H*, the strength of the liquid–wall interaction *ε*_LW_, and the crystal density *ρ*_W_.

**Fig. 2:**
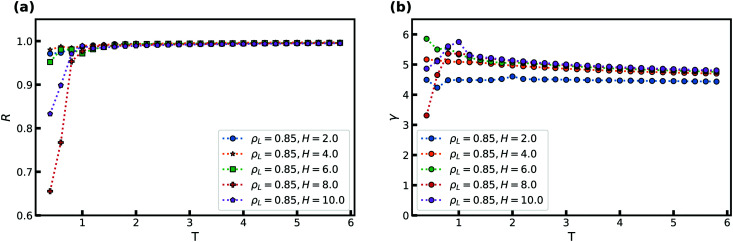
The correlation coefficient *R* and density-scaling exponent *γ* as a function of temperature *T* for several slit-pore widths *H* for the SCLJ liquid. The liquid density is *ρ*_L_ = 0.85, and the wall density is *ρ*_W_ = 1. (a) *R* as a function of temperature. (b) *γ* as a function of temperature.

Fig. 2 shows how *R* and *γ* (eqn (2) and (4)) vary with temperature for several slit-pore widths in the range *H* = [2; 10]. The confinement thus ranges from almost a single layer of liquid particles to more bulk-like conditions. The densities of the liquid and wall are kept constant with *ρ*_L_ = 0.85 and *ρ*_W_ = 1, respectively. For simplicity, we here and henceforth define the liquid density as *ρ*_L_ ≡ *N*/*AH*, where *A* is the exposed surface area of the crystal. We thus do not take into account any excluded volume near the walls when calculating the confined liquid density.^38^ As a reference, the bulk liquid has *R* ≈ 0.96 at the chosen liquid density and temperatures around unity.

The correlation coefficient *R* increases with temperature, and for temperatures above unity we find only a very weak dependence on *H* as close encounters start to play a big role. For low temperatures we find that the slit-pore is fully crystallized but depending on *H* negative pressures are also encountered. Negative pressure tends to break any strong correlation as the system wants to phase separate. In the previous smooth slit-pore study,^37^ a decrease in *R* was observed with smaller *H*. There were two effects in play for this breakdown: crystallization and the wall potential. Typically, two-phase coexistence lowers the correlation coefficient. In the bulk, for a SCLJ liquid the explanation for Roskilde simplicity is given in terms of an inverse power-law and linear potential, the latter of which adds to a constant when summing over the nearest neighbours. This linear potential will not match perfectly with the walls, compared to the liquid particles, and therefore also lowers the correlation coefficient. As *H* is lowered this effect becomes stronger as more particles feel the full effect of the walls. Here, we do not observe this strong effect for fcc walls.

The density-scaling exponent *γ* displays the opposite trend in Fig. 2(b) showing a monotonic decrease with temperature for all *H* (except for the negative pressure state points). *γ* is noted to increase with slit-pore width, but no theory currently exists for *γ*'s dependence on *H*.^28,64^ We find a possible slit-pore-width dependent plateau for *γ* between 4 and 5 which signifies that *γ* is also dependent on *H* in confined liquids. More investigations are nevertheless needed to determine if this *H*-dependent plateau truly exists and if it has a physical significance.

**Fig. 3:**
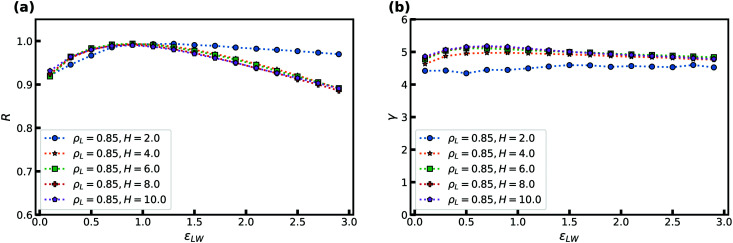
The correlation coefficient *R* and density-scaling exponent *γ* as a function of the strength of the liquid–wall interaction *ε*_LW_ for several slit-pore widths *H*. Temperature is fixed at *T* = 2, the liquid density is *ρ*_L_ = 0.85, and the wall density is *ρ*_W_ = 1. (a) *R* as a function of *ε*_LW_. (b) *γ* as a function of *ε*_LW_.

We now consider the effect of varying the attraction between the wall and the confined liquid particles. In doing so, we keep the wall–liquid cutoff fixed at *r*_c_ = 2.5*σ*_AW_ which should not affect our results much, given that the range of interaction of the wall is short for R-simple liquids in nanoconfinement.^65^ This was also confirmed by probing a cutoff at *r*_c_ = 5.0*σ*_AW_. Fig. 3 shows *R* and *γ* as a function of *ε*_LW_ at *T* = 2 and *ρ*_L_ = 0.85, again for several *H*. As the simulations use *ε*_AA_ = 1, for *ε*_LW_ > 1 we have an “attractive” wall, and when *ε*_LW_ < 1 it becomes a “repulsive” wall with respect to the interactions between the liquid particles.

Fig. 3(a) shows that *R* depends significantly on *ε*_LW_. For the larger *H*, a decrease in the correlation coefficient is found with increasing attraction as the wall potential plays a bigger role. This trend is also observed with decreasing strength as the system becomes more gas like. For *H* = 2, the correlation coefficient *R* increases with *ε*_LW_ as the slit-pore crystallizes fully (the solid, in general, has higher *R* than the liquid phase^28^). The density-scaling exponent in Fig. 3(b) displays a behavior that mimics that of the correlation coefficient, but the maximum is displaced to lower values of *ε*_LW_.

The highest value of *R*, and therefore the maximum, is expected to appear when the wall particles are most similar to the liquid particles, which here means around *ε*_LW_ = 1. We observe that the maximum occurs slightly to the left for *R* and is consistent with this expectation but for much lower values for *γ*. We currently have no explanation for this observation.

**Fig. 4:**
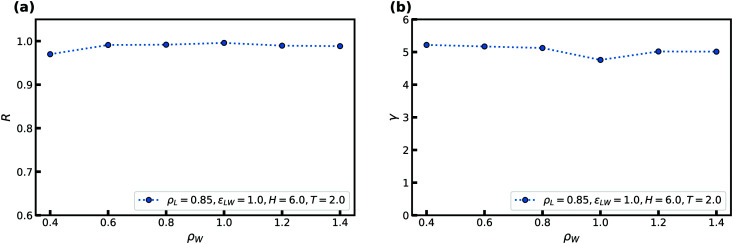
The correlation coefficient *R* and density-scaling exponent *γ* as a function of crystal density *ρ*_W_ at *T* = 2, *H* = 6, and *ε*_LW_ = 1. The liquid density is *ρ*_L_ = 0.85. (a) *R* as a function of *ρ*_W_. (b) *γ* as a function of *ρ*_W_.

Another means to probe the coupling between the confined liquid and the walls is to change the density of the fcc walls *ρ*_W_. In Fig. 4 we show the correlation coefficient and density-scaling exponent as a function of the density of the crystal. The liquid density is kept constant at *ρ*_L_ = 0.85 and *H* = 6. We note that changing the crystal density does not keep a constant energy density, and hence particles will automatically interact more strongly with the walls as a whole for higher densities and the opposite for lower densities.

We observe for low *ρ*_W_ that *R* decreases when the density of the wall is reduced, though it remains well above 0.90. This effect can be attributed to particles penetrating into the walls (not shown). For high *ρ*_W_ the correlation coefficient remains virtually constant. Almost no effect on *γ* is noted in Fig. 4(b) for both low and high *ρ*_W_.

### B. Isomorph invariance of reduced density profiles and dynamics

We now turn to isomorphs in the nanoconfined system. To this end, we consider the behaviour along an isomorph and contrast it with an isochore at a density *ρ*_L_ = 0.85. Isomorphs in the SCLJ liquid were generated by the direct-isomorph-check method (see Section III). We consider two different slit-pore widths, one with *H* ≈ 6 and one with *H* ≈ 10; recall that *H* is adjusted with the liquid density along the isomorphs. These two distances span a strong and medium confined liquid.

**Fig. 5:**
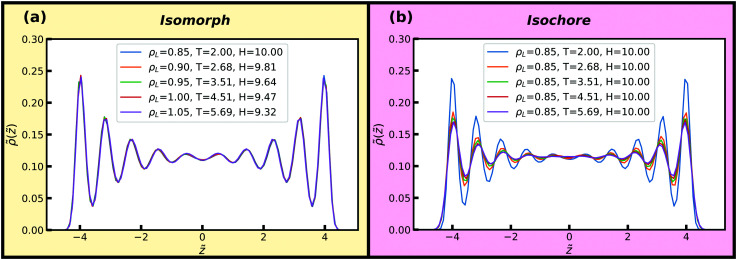
Reduced density profiles of the liquid particles perpendicular to the walls along an isomorph and an isochore. Excellent invariance is seen along the isomorph but not along the isochore, with significant changes in all peak heights. (a) Isomorph. (b) Isochore.

Density profiles perpendicular to the walls along an isomorph with *H* ≈ 10 are shown in Fig. 5(a). For comparison, results for an isochore with the same temperature variation are given in Fig. 5(b). From this point forward a yellow figure background denotes data obtained along an isomorph, and a pink figure background denotes data obtained along an isochore.

We find that the density profiles to a good approximation are invariant along the isomorph with a 24% density increase, whereas this is not the case along the isochore in Fig. 5(b). For the isochore, significant changes in all peak heights with temperature are observed, in particular for the layer closest to the wall indicating pre-crystallisation even at *H* = 10. This pre-crystallization is, however, nicely preserved on the isomorph.

**Fig. 6:**
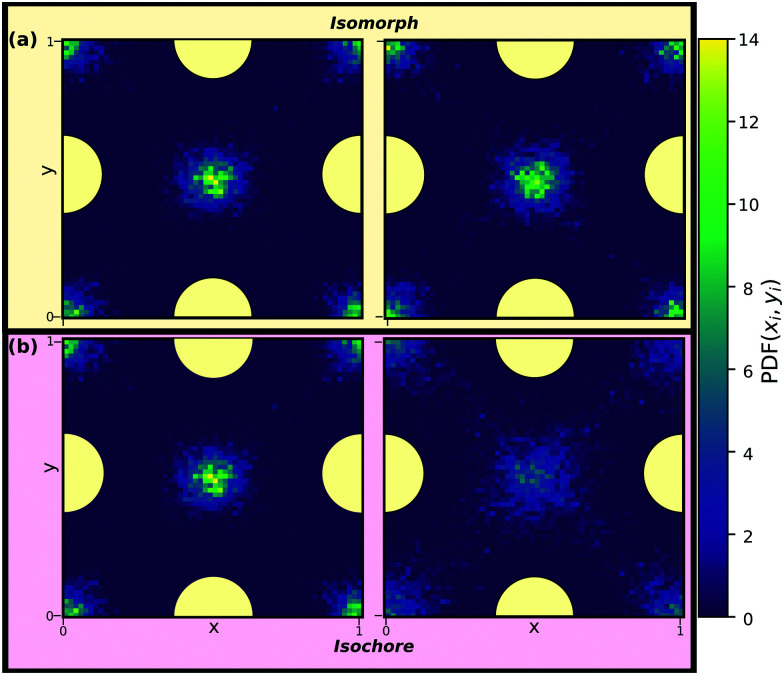
Reduced in-plane density profiles of the liquid particles in the layer closest to the walls, *i.e.* |*z̃*| ≈ 4, for the first (left) and last (right) state points of the (a) isomorph and (b) isochore of Fig. 5. One unit cell of the crystalline walls is shown; the yellow circles indicate fcc wall particles.

Fig. 6 displays the in-plane density profiles in the layer closest to the walls, *i.e.*, for the layer around |*z̃*| ≈ 4, for the first and the last state points of Fig. 5. The density profile is shown for one unit cell of the crystalline walls. The isomorph (a) shows excellent scaling with the liquid particles situated in between the wall particles and exhibits very little change in the density profile whereas the isochore (b) displays a density field that is increasingly smeared out as *T* is increased, confirming the pre-crystallisation. Similar behavior is observed for the remaining layers (not shown).

**Fig. 7:**
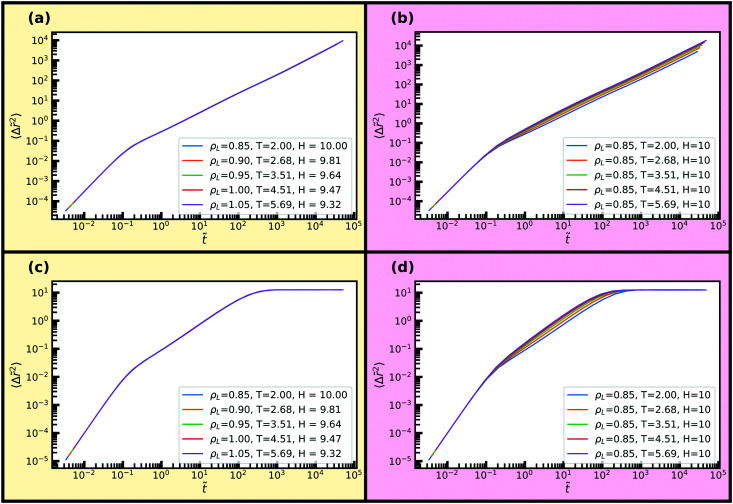
Reduced average mean-square displacements parallel and normal to the walls along the isomorph and isochore of Fig. 5. (a) Isomorph, parallel dynamics. (b) Isochore, parallel dynamics. (c) Isomorph, normal dynamics. (d) Isochore, normal dynamics.

Dynamical properties such as the mean-squared displacement (MSD) or the intermediate scattering function are also invariant along an isomorph, and we now examine how well this behaviour holds in nanoconfinement. To do so, we consider the reduced MSD parallel and normal to the walls as a function of reduced time in Fig. 7 along the same isomorph and isochore as before. The MSD is averaged over the entire slit-pore. We find excellent invariance along the isomorph and visible variation for the isochore. For the bulk liquid, at these high temperatures, one would see a similar scaling in comparison to the isochore; the differences becoming more pronounced with the degree of supercooling.^32^

### C. Isomorph invariance of higher-order structures

Two-point spatial correlation functions, such as the radial distribution function, have been shown to a good approximation to be invariant along isomorphs in simulations of both bulk and smooth-wall confined systems.^30,34,37,66^ Higher-order structural correlations have until very recently^67^ been investigated to a lesser extent and could well be less invariant than the two-body correlation functions as the isomorph theory is approximate. In particular, geometric motifs, so-called locally favoured structures (LFS) such as the bicapped square antiprism^68,69^ have been seen to vary significantly along isomorphs in supercooled KABLJ.^39^

**Fig. 8:**
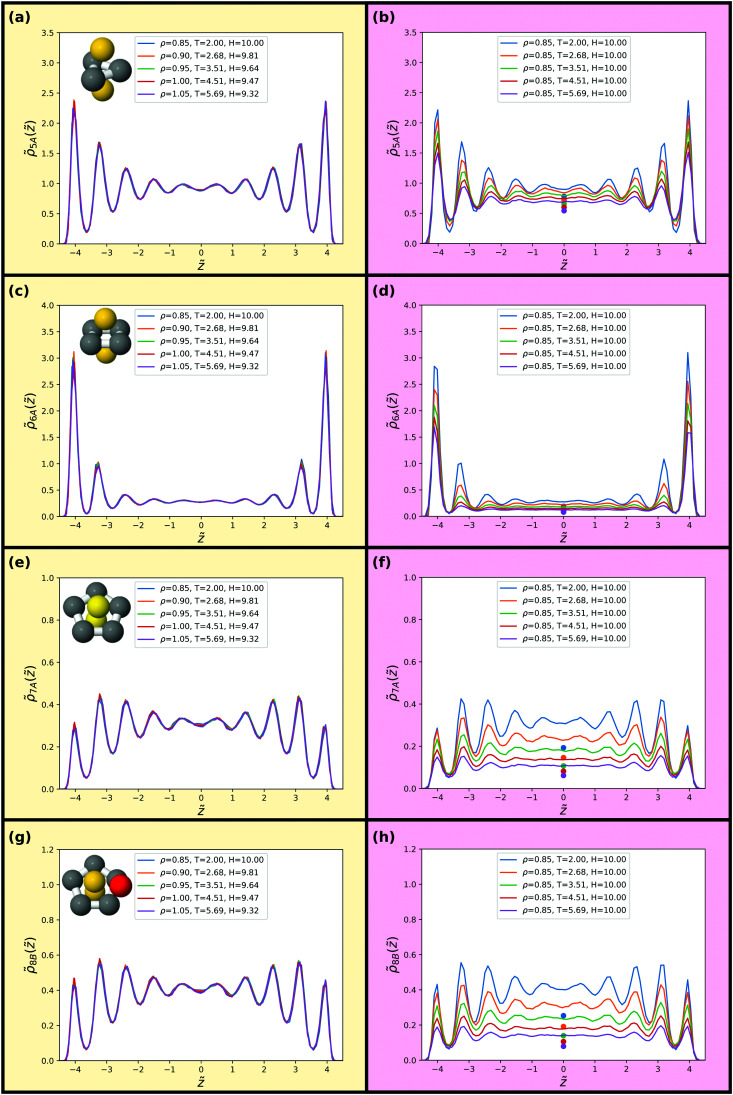
Populations of minimum energy clusters along the previously studied isomorph (left) and isochore (right). The minimum energy clusters considered in each case are illustrated in the corresponding panels. We consider the 5-membered triangular bipyramid in (a) and (b), the *m* = 6 octahedron in (c) and (d), the *m* = 7 pentagonal bipyramid in (e) and (f) and the *m* = 8 cluster with *C*_s_ symmetry in (g) and (h). The data points in (b, d, f and h) give bulk isochore values at the same density and temperature.

**Fig. 9:**
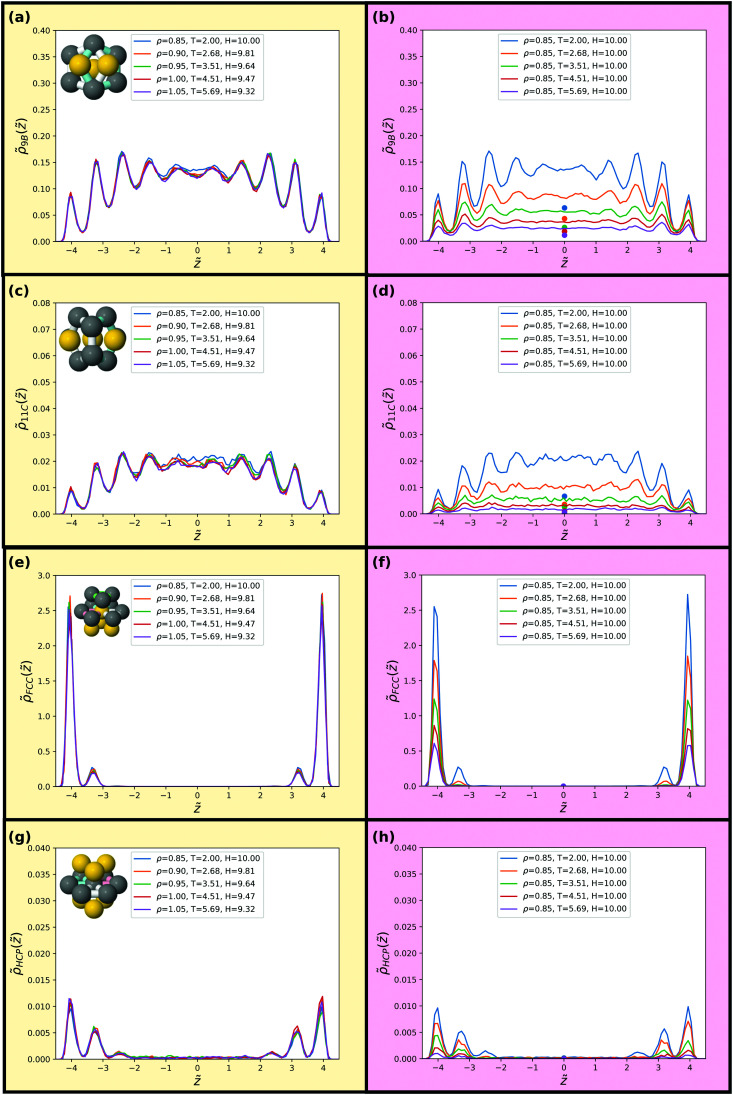
Populations of minimum energy clusters along the previously studied isomorph (left) and isochore (right). The minimum energy clusters considered in each case are illustrated in the corresponding panels. We consider the 9-membered *C*_2v_ symmetric cluster in (a) and (b), the *m* = 11 *C*_2v_ symmetric cluster in (c) and (d), the fcc local crystalline environment in (e) and (f) and the hcp local crystalline environment in (g) and (h). The data points in (b, d, f and h) give bulk isochore values at the same density and temperature.

As a final probe of isomorphs for the confined SCLJ liquid we investigate in Fig. 8 and 9 invariance of higher-order structures. The liquids here are not supercooled much, and thus rather than a locally favoured structure we instead consider minimum energy clusters of 5 ≤ *m* ≤ 13 particles for these systems.^69,70^ These minimum energy clusters typically include the LFS, although the latter often exhibit a specific symmetry^71^ while the minimum energy clusters for each system exhibit a range of symmetries. In addition, we consider populations of the hcp and fcc crystalline structures. The minimum energy structures are identified by the topological cluster classification.^40^ The eight structures of the SCLJ system are depicted in the figures (top left in each panel).^40,70,72^

We find that the distribution of clusters is, to an excellent approximation, invariant along the isomorph but not along the isochore. For all structures on the isochore the variation is around a factor of two with hardly any visible variation along the isomorph. Very minor deviations are, however, noted for the 9B and 11C structures along the isomorph. We emphasize that the probing of these structures does not imply relevance of these structures for the dynamics of the liquid but merely used for testing invariance of higher-order structures along an isomorph which are predicted to be invariant.

While the structures considered exhibit very little change along the isomorph, the changes between the behaviour of the different clusters is notable in itself. We can identify three regimes: small amorphous clusters, larger amorphous clusters and crystalline structures. As shown in Fig. 8(a) and (c), the smaller amorphous clusters, the triangular bipyramid 5A and octahedron 6A, largely follow the density profiles illustrated in Fig. 5. Larger amorphous clusters, beginning with the pentagonal bipyramid 7A, have a degree of fivefold symmetry and their population is suppressed close to the wall (see Fig. 8(e), (g) and 9(a), (c)). Interestingly, this is not observed in the case of a free interface where the cluster population is rather slaved to the density profile.^73^ A rather different behaviour is found for the crystalline structures, where the layer by the wall has a high population of particles in a crystalline environment, but the population in the middle of the slit is very small (Fig. 9(e) and (g)).

We compare these results with bulk populations of the corresponding clusters for the same temperature and density as shown by the colored data points in Fig. 8 and 9. The data points are plotted for the isochore data but may be taken to be representative of the isomorph data on the left hand side of the figure. Even in the centre of the slit, the results show a strong enhancement of cluster population with respect to the bulk in all cases except for the hcp and fcc crystals, whose population in the centre of the slit is negligible. This is remarkable, given that for a free liquid–vapour interface, cluster populations reach their bulk value around a diameter from the interface.^73^ Further work is called for to understand this unexpected increase in higher-order structure in confinement.

In the ESI† we present results for an isomorph with *H* ≈ 6. We find excellent scaling of density profiles and mean-square displacements. To conclude on the SCLJ liquid, we find that isomorphs survive into heavily confined systems with fcc crystalline walls and have excellent scaling also for higher-order structures.

## V. Kob–Andersen binary Lennard-Jones mixture

We now turn to investigate similar quantities for the KABLJ mixture. Although being a binary mixture prized for its glassforming ability, the KABLJ mixture is prone to crystallization in the bulk.^74^ The mechanism for crystallization in the bulk occurs through the formation of fcc (and hcp) nuclei of the majority A species.^74^ We find that the KABLJ mixture like the SCLJ liquid in confinement also (pre)crystallizes and suggests that heterogeneous nucleation at the walls (which are patterned as an fcc structure) is a powerful mechanism for the KABLJ mixture, as also seen for other simple liquids.^3,4,50^

### A. Variation in the correlation coefficient *R* and density-scaling exponent *γ*

**Fig. 10:**
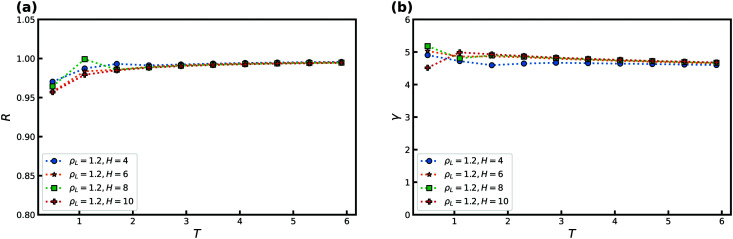
*R* and *γ* as a function of temperature *T* for several slit-pore widths *H* for the KABLJ mixture at *ρ*_L_ = 1.2 and *ρ*_W_ = 1. (a) *R* as a function of temperature. (b) *γ* as a function of temperature.

Fig. 10 displays *R* and *γ* as a function of temperature for several slit-pore widths *H* for the KABLJ mixture at *ρ*_L_ = 1.2 and *ρ*_W_ = 1 with *ε*_AW_ = 1, and *ε*_BW_ = 0.707. The bulk correlation coefficient at this density is *R* ≈ 0.96. As for the SCLJ liquid we find an increase in *R* and a decrease in *γ* with temperature. For the KABLJ mixture, however, at low temperatures we do not find full crystallization as one expects for a reasonable glassformer. We find that the precrystallization consists only of A-particles.

**Fig. 11:**
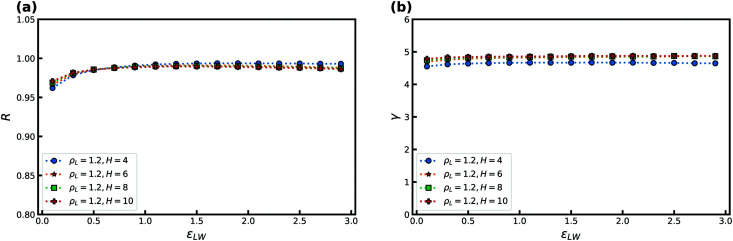
*R* and *γ* as a function of the liquid–wall interaction strength *ε*_LW_ for several slit-pore widths *H*. Temperature is fixed at *T* = 2.5, and the liquid density is *ρ*_L_ = 1.2. (a) *R* as a function of *ε*_LW_. (b) *γ* as a function of *ε*_LW_.

Next, we consider the effect of changing the liquid–wall interaction strength *ε*_LW_ in Fig. 11. A weak maximum is noted for both *R* and *γ* and is displaced slightly away from the value of *ε*_AW_ = 1. Good correlation is noted for the entire range of attractions. For the KABLJ mixture the effect of the crystal density *ρ*_W_ was not investigated.

### B. Isomorph invariance of reduced density profiles and dynamics

**Fig. 12:**
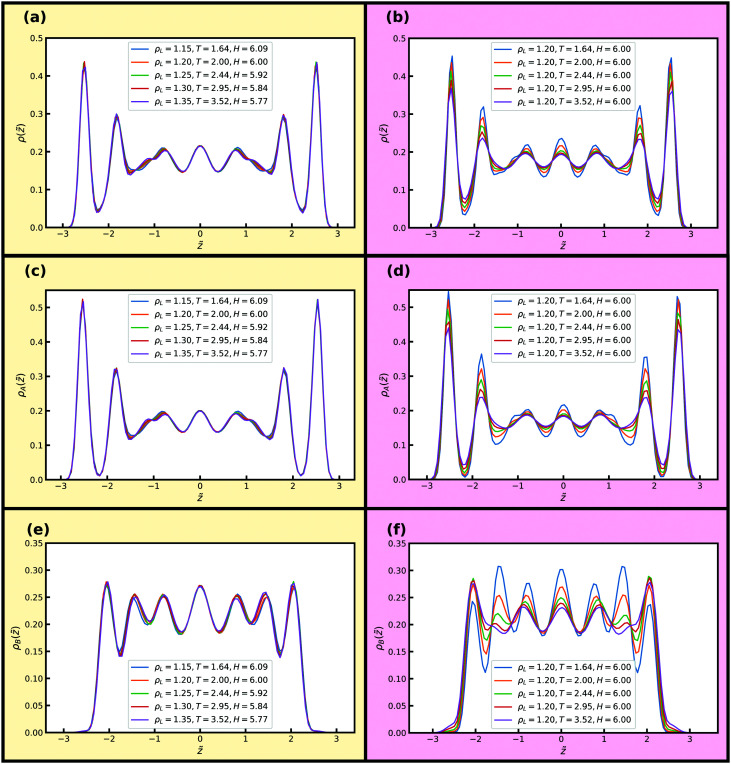
Reduced density profiles of the liquid particles perpendicular to the walls along an isomorph and an isochore. (a) Total density profile, isomorph. (b) Total density profile, isochore. (c) A-particle density profile, isomorph. (d) A-particle density profile, isochore. (e) B-particle density profile, isomorph. (f) B-particle density profile, isochore.

For the KABLJ mixture we also investigate two isomorphs with *H* ≈ 6 and *H* ≈ 10 to facilitate comparison with the SCLJ liquid in the previous section. Fig. 12(a), (c) and (e) show reduced normal density profiles for the total and partial densities along an isomorph with 17% density increase and *H* ≈ 6. Fig. 12(b), (d) and (f) show the corresponding quantities along an isochore. Good invariance is noted along the isomorph but not along the isochore where again significant changes in the peak heights are noted for all density profiles, in particular for the B-particle density profiles (Fig. 12(f)).

**Fig. 13:**
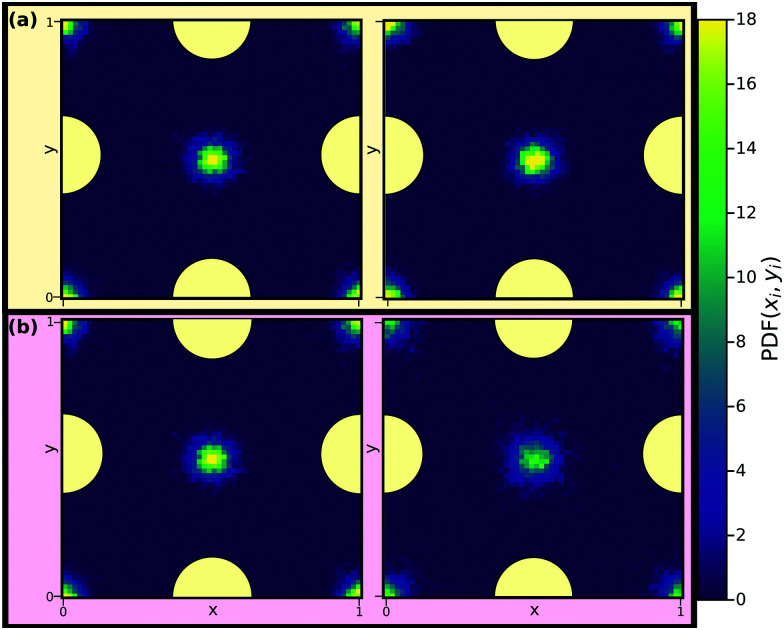
Reduced in-plane total density profiles of the liquid particles in the layer closest to the walls, *i.e.* |*z̃*| ≈ 2.7, for the first (left) and last (right) state points along the isomorph (a) and the isochore (b) of Fig. 12. One unit cell of the crystalline walls is shown; the yellow circles indicating fcc wall particles.

Fig. 13 shows reduced in-plane total density profiles for the layer closest to the wall, *i.e.* |*z̃*| ≈ 2.7, for the first and last state points of the same isomorph and isochore. For the KABLJ mixture the in-plane density profile does not seem to show a strong deterioration along the isochore as found for the SCLJ liquid, which could be anticipated from the height variation of the first peak in Fig. 12(b). Nevertheless the invariance is still visually worse than that of the isomorph.

**Fig. 14:**
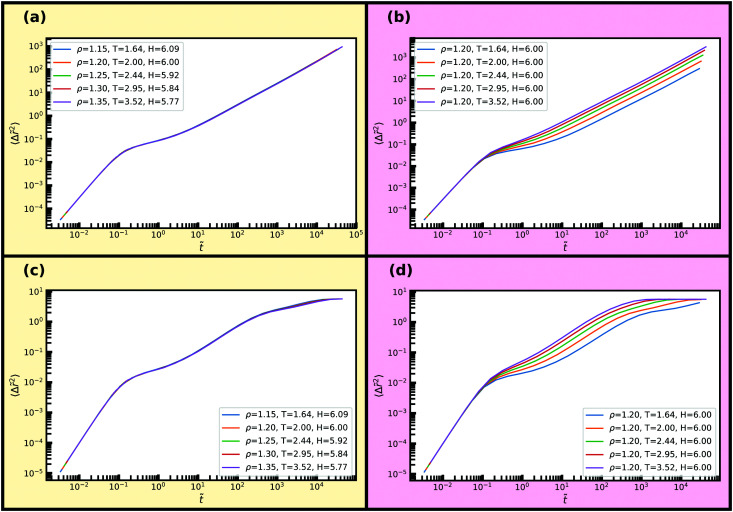
Reduced A-particle mean-square displacement parallel and normal to the walls averaged over the entire slit-pore along an isomorph and an isochore. (a) Isomorph, parallel dynamics. (b) Isochore, parallel dynamics. (c) Isomorph, normal dynamics. (d) Isochore, normal dynamics.

For the reduced normal and parallel A-particle MSDs in Fig. 14 almost perfect scaling is observed along the isomorph while approximately a decade deviation in diffusion coefficient is observed for the isochore. These deviations in MSD are similar to what is seen for supercooled bulk liquids.^30,34^ We find for the B-particles a very similar scaling behaviour (not shown).

### C. Isomorph invariance of higher-order structures

**Fig. 15:**
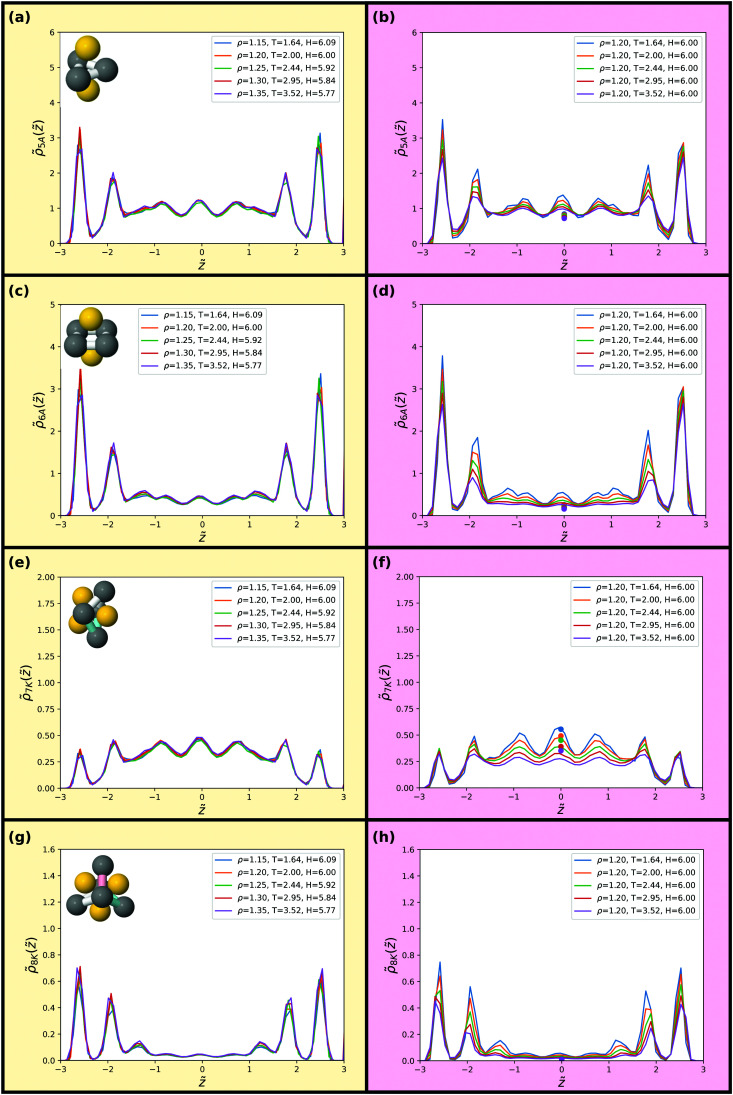
Populations of minimum energy clusters along the previously studied isomorph (left) and isochore (right). The minimum energy clusters considered in each case are illustrated in the corresponding panels. We consider the 5-membered triangular bipyramid in (a) and (b), the *m* = 6 octahedron in (c) and (d), the *m* = 7 polytetrahedron in (e) and (f) and the *m* = 8 pyramidal geometry in (g) and (h). The data points in (b, d, f and h) give bulk isochore values at the same state points.

**Fig. 16:**
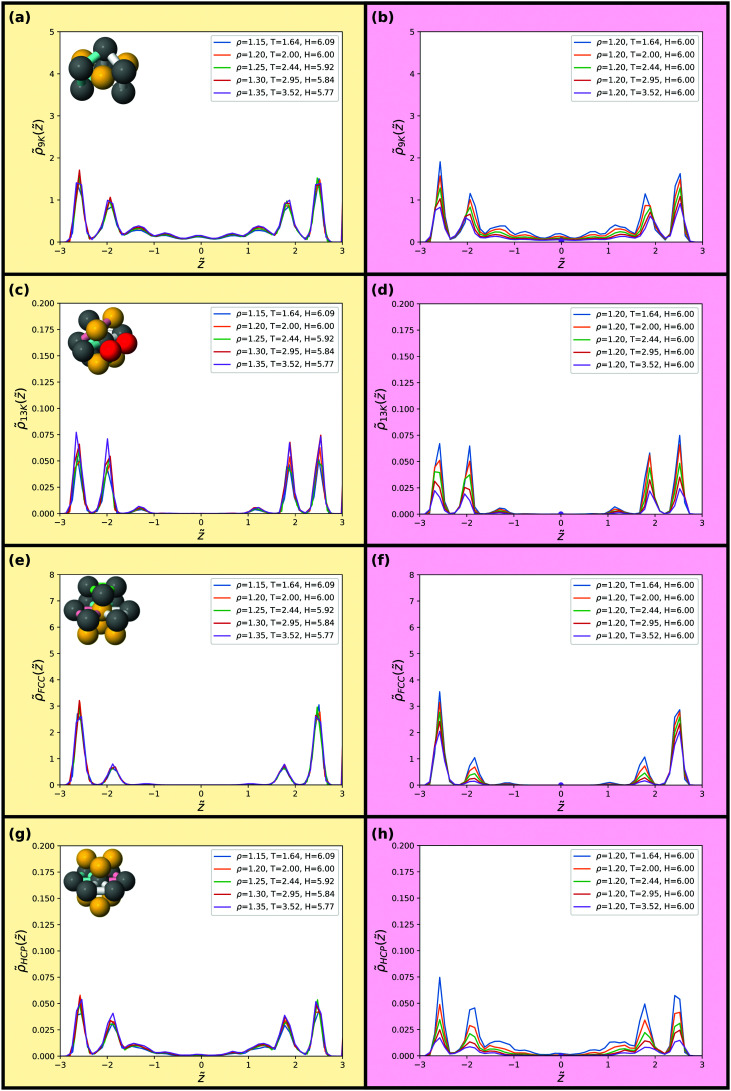
Populations of minimum energy clusters along the previously studied isomorph (left) and isochore (right). The minimum energy clusters considered in each case are illustrated in the corresponding panels. We consider the 9-membered triangular antiprism in (a) and (b), the *m* = 13 polytetra-octahedron (c) and (d), the fcc local crystalline environment in (e) and (f) and the hcp local crystalline environment in (g) and (h). The data points in (b, d, f and h) give bulk isochore values at the same state points.

Finally, we consider invariance of selected minimum energy clusters for the KABLJ mixture^40,69^ in Fig. 15 and 16, reaching a similar conclusion as for the SCLJ liquid with excellent invariance along the isomorph but not along the isochore, the latter showing around a factor of two variation in almost all structures. Although, the bicapped square antiprism (11A) structure has been shown to correlate reasonably to the dynamics of the KABLJ system^69,75–77^ we find very few bicapped square antiprisms at the higher temperatures considered here (the onset temperature for glassy dynamics, at which the bicapped square antiprism becomes popular, is around *T* ≈ 1.0), and 11A is therefore not included in the figures.

It is quite remarkable that the minimum energy clusters of the KABLJ system^40,69^ show such a good invariance, even for higher-order correlations, given that previous work showed a significant discrepancy in precisely the same system,^39^ although at the lower temperature *T* = 1.0. The results presented here are at a higher temperature (*T* > 2.0), the magnitude of the discrepancy and its rather weak temperature dependence leads one to speculate whether the confinement may somehow influence the agreement.

In comparing with the bulk values (data points in Fig. 15 and 16 right hand side), we see similar to the SCLJ system (Fig. 8 and 9) that in many cases the cluster population even at the centre of the slit is markedly higher than the bulk liquid at the same state point. However, this is not universally the case here, as the *m* = 7 polytetrahedron (7K) in fact seems to sit right on the confined data for some state points, while at higher temperature the bulk population seems higher than the confined system. The reasons for the change in behaviour of this structure and, as noted above, why the minimum energy clusters typically have a reduced population with respect to the bulk is an interesting topic for future work. In the ESI,† we compare bulk KABLJ data with the smooth wall simulations of ref. 37. We find here that the agreement is better and hence the templating of the walls plays a role.

We provide in ESI† figures for an isomorph with *H* ≈ 10. Similar conclusions are reached, showing again good invariance along the isomorph.

## VI. Summary and outlook

Isomorphs are curves in the thermodynamic phase diagram of R-simple liquids along which structure and dynamics in reduced units to a good approximation are invariant. However, nanoconfined liquids show strikingly different behavior from bulk liquids in terms of their structure and dynamics.^4^ It is therefore not obvious that concepts demonstrated in the bulk apply also to confined liquids. Extending the isomorph framework to nanoconfined liquids is important as theories for nanoconfined liquids have been slower to develop due to the added complexity.

A previous study^37^ explored the existence of isomorphs in nanoconfined liquids using a smooth slit-pore geometry and found that isomorphs do survive under confinement. Here, we have studied the effect of introducing highly inhomogenous density profiles both parallel and perpendicular to the walls by applying more realistic crystalline fcc walls. The effect of the wall-to-wall distance *H*, the strength of liquid–wall interactions *ε*_LW_, and the crystal density *ρ*_W_ were explored on two bulk R-simple liquids: the SCLJ liquid and the KABLJ mixture.

Although strong inhomogeneities occur in this type of confinement, we found that R-simple liquids and isomorphs survive down to a few particle diameters confinement. More specifically, along isomorphs we probed density profiles, normal and parallel mean-squared displacements, as well as higher-order structures using the topological cluster classification algorithm. Even for higher-order correlations of populations of minimum energy clusters up to 13 particles, we found excellent invariance along the isomorphs. This is notable, as in the bulk, albeit at lower temperatures, considerable deviation was found for higher-order structures even when the two-point structure scaled well.^39^ Curiously, in many (but not all) of these clusters, the population in the confined system, even at the centre, is markedly higher than that in the bulk for the same state point. This is remarkable, given that in the case of a free liquid–vapour interface, the cluster population reaches its bulk value within around a diameter of the interface;^73^ we found that this is likely connected to the templating of the walls. This curiosity should be investigated in future work.

We conjecture from the current study that even very complicated confinements, *e.g.*, carbon nanotubes, can exhibit Roskilde simplicity and thus also their associated scaling laws, such as Rosenfeld excess-entropy scaling. This provides an important simplification of the phase diagram and valuable insights into the structures and dynamics of confined liquids. In this connection, an intriguing path for further research is the development of equations of state for confined liquids using the isomorph theory.^31^ Furthermore, it is known that thermostatting the walls can affect the structure of the confined liquid to a great extent^45,46^ and hence might also affect Roskilde simplicity. This would have to be studied in more detail in the future.

## Conflicts of interest

There are no conflicts of interest to declare.

## Supplementary Material

SM-017-D1SM00233C-s001
