# Supplementary material for: Isomorphs in nanoconfined liquids
Source: Soft Matter. 2021 Sep 13;17(38):8662–77. doi: 10.1039/d1sm00233c (PMC8494272; doi:10.1039/d1sm00233c)
Supplement: SM-017-D1SM00233C-s001 [file SM-017-D1SM00233C-s001.pdf]

Electronic Supplementary Material (ESI) for Soft Matter.
This journal is © The Royal Society of Chemistry 2021

Supplementary Material for "Isomorphs in nanoconfined liquids"

Benjamin M. G. D. Carter,[1,2] C. Patrick Royall,[1,3,4] Jeppe C. Dyre,[5] and Trond S. Ingebrigtsen[5]
[1] *H.H. Wills Physics Laboratory, Tyndall Avenue, Bristol, BS8 1TL, UK.*
[2] *Bristol Centre for Functional Nanomaterials, Tyndall Avenue, Bristol, BS8 1TL, UK.*
[3] *School of Chemistry, University of Bristol, Cantock's Close, Bristol, BS8 1TS, UK*
[4] *Centre for Nanoscience and Quantum Information, Tyndall Avenue, Bristol, BS8 1FD, UK*
[5] *Department of Science and Environment, Roskilde University, Postbox 260, DK-4000 Roskilde, Denmark*

I. SINGLE-COMPONENT LENNARD-JONES LIQUID

The figures given below summarize selected quantities for an isomorph with $H \approx 6$ for the SCLJ liquid.

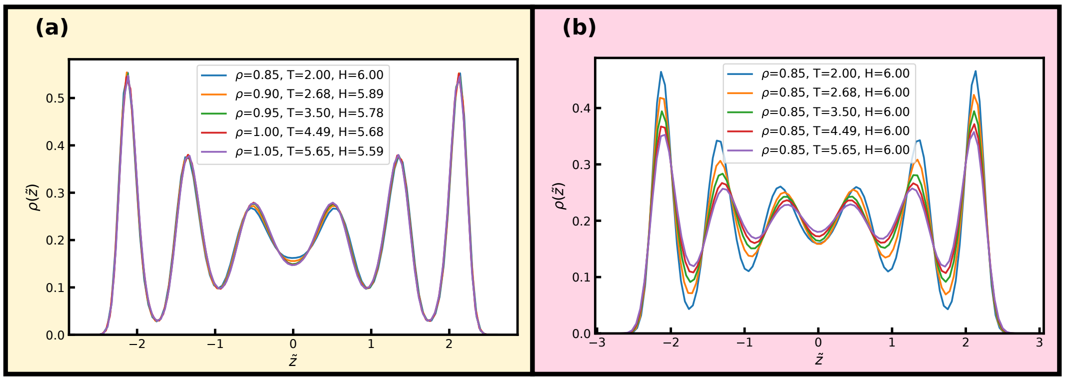

FIG. 1: Reduced density profiles of the liquid particles perpendicular to the walls along an isomorph and an isochore. (a) Isomorph. (b) Isochore.

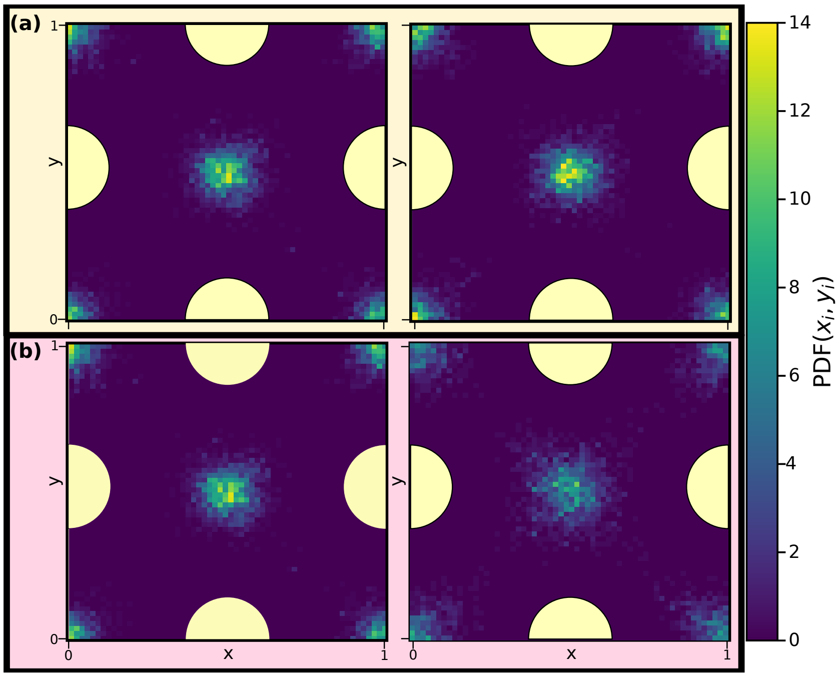

FIG. 2: Reduced in-plane density profiles of the liquid particles in the layer closest to the walls, i.e. $|\tilde{z}| \approx 2$, for the first and last state points along the isomorph (top) and isochore (bottom) of Fig. 1. One unit cell is shown with the yellow circles indicating fcc wall particles.

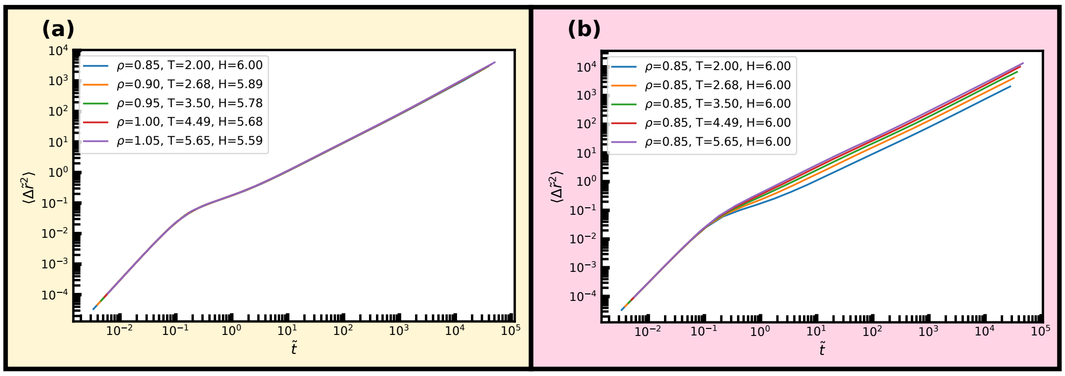

FIG. 3: Reduced average mean-square displacements parallel to the walls along the same isomorph and isochore. (a) Isomorph. (b) Isochore.

II. KOB-ANDERSEN BINARY LENNARD-JONES MIXTURE

The figures given below summarize selected quantities for an isomorph with $H \approx 10$ for the KABLJ mixture.

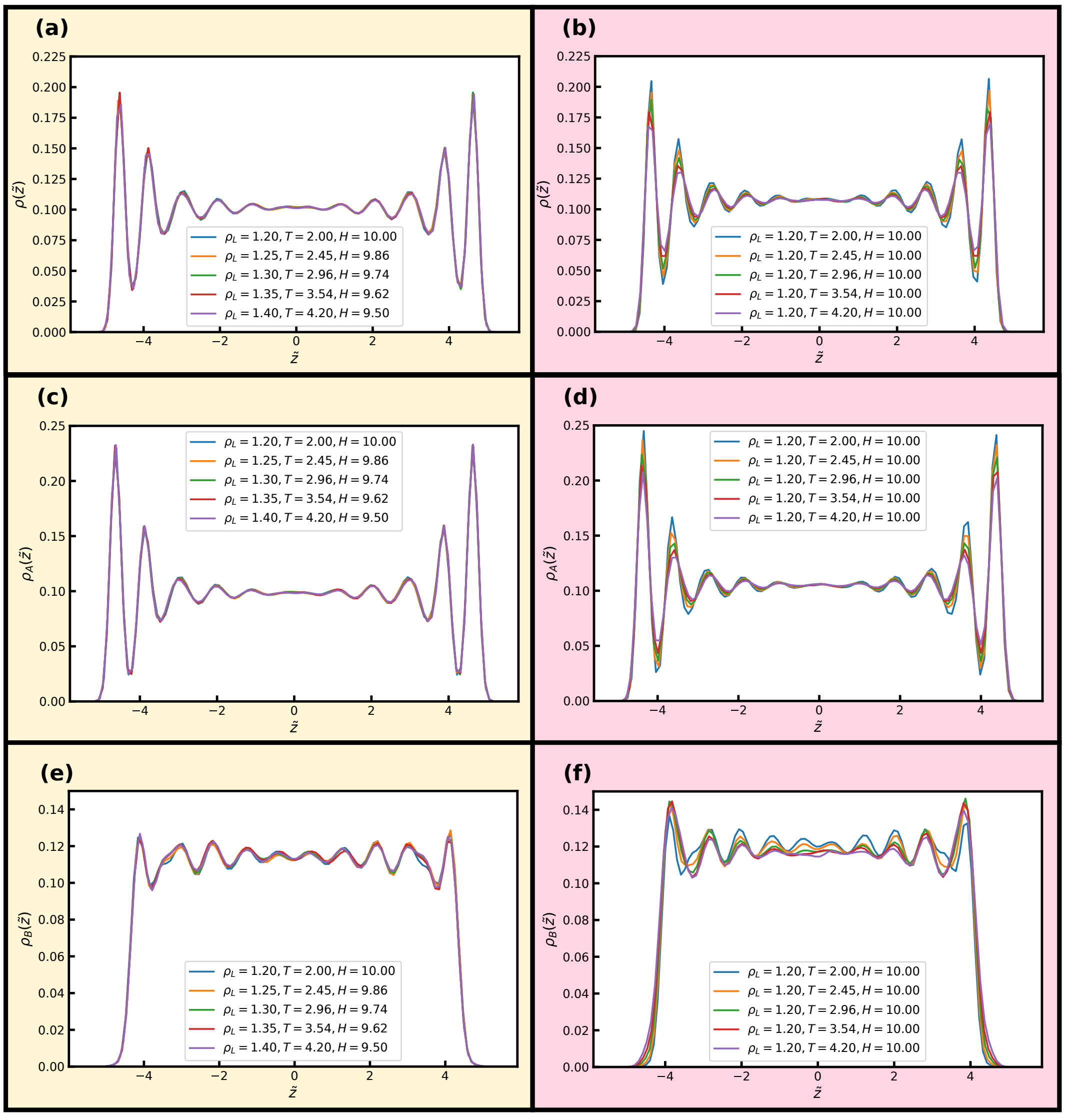

FIG. 4: Reduced density profiles of the liquid particles perpendicular to the walls along an isomorph and an isochore. (a) Total density profile, isomorph. (b) Total density profile, isochore. (c) A-particle density profile, isomorph. (d) A-particle density profile, isochore. (e) B-particle density profile, isomorph. (f) B-particle density profile, isochore.

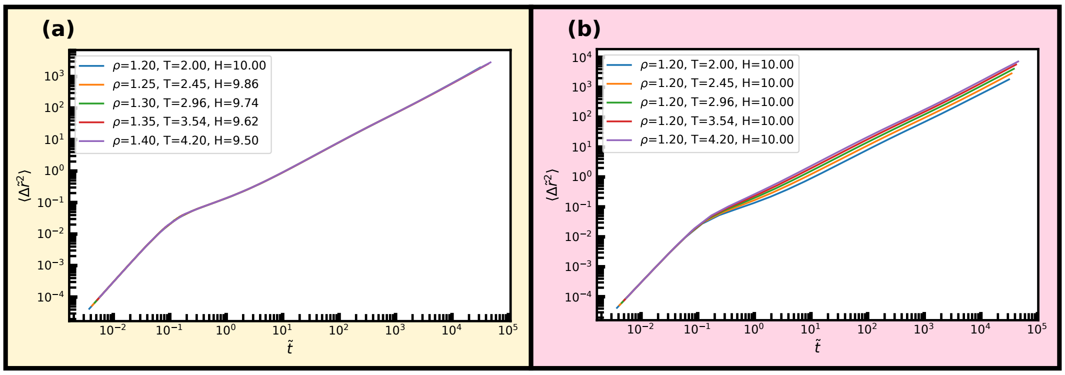

FIG. 5: Reduced average A-particle mean-square displacements parallel to the walls along the same isomorph and isochore. (a) Isomorph. (b) Isochore.

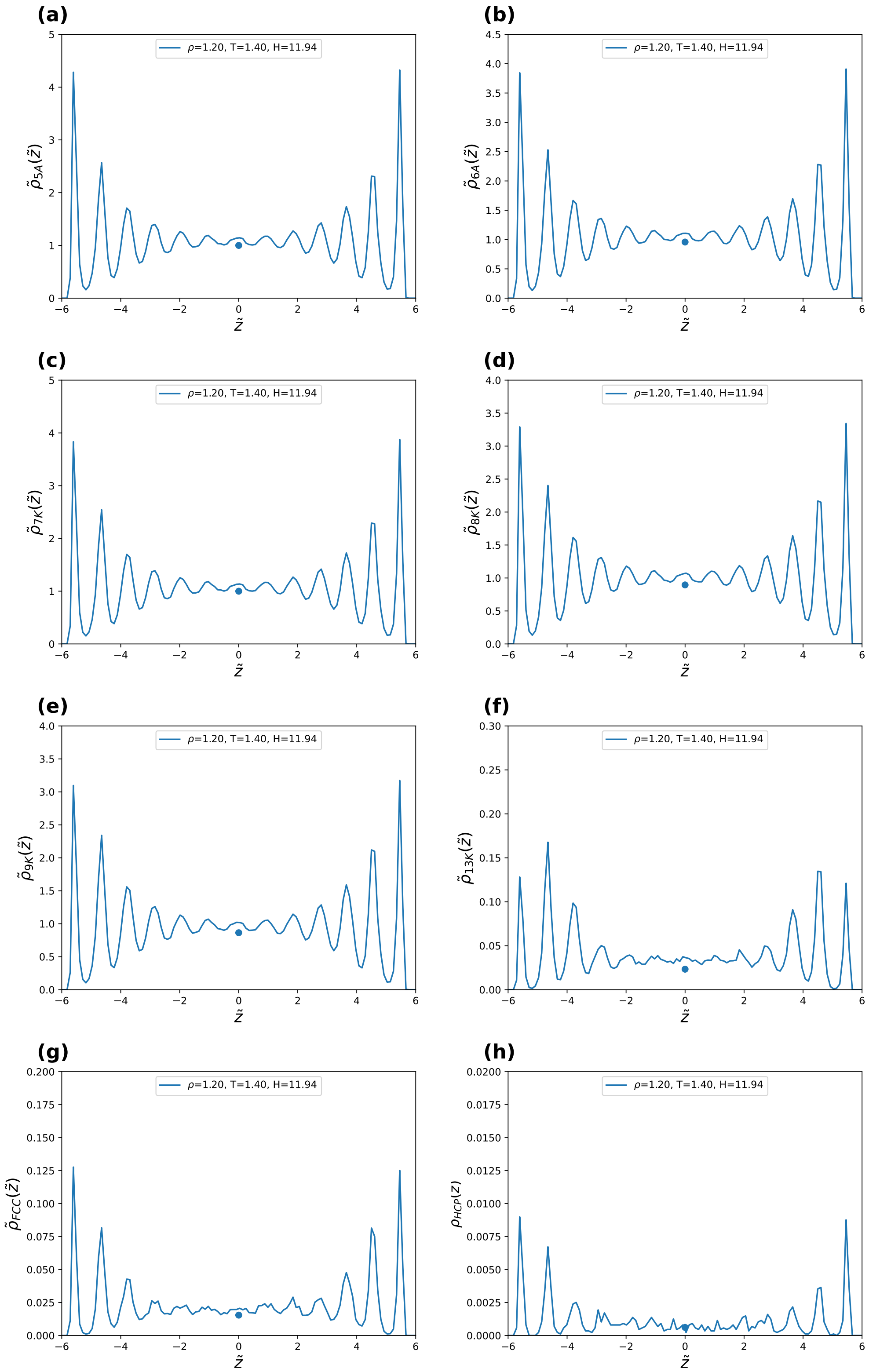

FIG. 6: Populations of minimum energy clusters for a smooth wall KABLJ mixture (see the main text for a description). The data points give bulk isochore values at the same state points.
